# Supplementary material for: Behavioral and Physiological Reactions to a Sudden Novel Object in the Weanling Horse: Quantitative Phenotypes for Future GWAS
Source: Genes (Basel). 2023 Feb 26;14(3):593. doi: 10.3390/genes14030593 (PMC10048014; doi:10.3390/genes14030593)
Supplement: Supplementary file 1 [file genes-14-00593-s001.zip › genes-2193128-supplementary.pdf]

Supplementary Table S1. Age and sex measures for four foal crop years.

Foal Crop	Tested Mean Age	Minimum Age	Maximum Age	Number Females	Number Males
**2014**	255.0	212	299	10	5
**2015**	244.0	188	287	11	7
**2016**	253.0	213	287	9	10
**2017**	265.0	217	315	11	11
